# Knowledge, Attitude, and Practices Regarding HIV and TB Among Homeless People in Tehran, Iran

**DOI:** 10.15171/ijhpm.2017.129

**Published:** 2017-11-07

**Authors:** Fahimeh Bagheri Amiri, Amin Doosti-Irani, Abbas Sedaghat, Noushin Fahimfar, Ehsan Mostafavi

**Affiliations:** ^1^Urology and Nephrology Research Center, Shahid Beheshti University of Medical Sciences, Tehran, Iran.; ^2^Department of Epidemiology, School of Public Health, Hamadan University of Medical Sciences, Hamadan, Iran.; ^3^Department of Epidemiology and Biostatistics, Research Centre for Emerging and Reemerging Infectious Diseases, Pasteur Institute of Iran, Tehran, Iran.; ^4^Blood Transfusion Research Center, High Institute for Research and Education in Transfusion Medicine, Tehran, Iran.; ^5^Center for Communicable Disease Control, Ministry of Health and Medical Education, Tehran, Iran.; ^6^Department of Epidemiology and Biostatistics, School of Public Health, Tehran University of Medical Sciences, Tehran, Iran.; ^7^HIV/STI Surveillance Research Center, and WHO Collaborating Center for HIV Surveillance, Institute for Futures Studies in Health, Kerman University of Medical Sciences, Kerman, Iran.

**Keywords:** Homeless, Tehran, KAP Study, HIV, Tuberculosis

## Abstract

**Background:** Homeless people are at high risk of HIV and tuberculosis (TB) infection due to living in poor sanitary conditions and practicing high-risk behavior. The aim of this study is to assess the knowledge, attitude, and practice (KAP) of homeless people in Tehran regarding TB and HIV.

**Methods:** Using a convenience sampling, we performed a cross-sectional study on homeless people in Tehran from June to August 2012. Participants aged 18-60 years having at least 10 days of homelessness in the preceding month to the study period were included. All required data were collected through face-to-face interviews conducted using a researcherdesigned questionnaire. Each score in KAP of TB and HIV was separately divided by the maximum score and multiplied by 100 to attain percentage scores. The mean scores were compared using analysis of variance (ANOVA) and student’s t test. A Tukey test was used for post hoc analysis and two-by-two comparisons.

**Results:** In this study, 593 participants consisting of 513 men and 80 women were included. The mean age of the participants was 41.74 ± 0.45 years. Moreover, the total mean score of KAP toward HIV was 79.24 (95% CI: 77.36, 81.12), 57.13 (95% CI: 55.12, 59.14), and 21.14 (95% CI: 18.35, 23.93), respectively. The total mean score of knowledge and practice regarding TB was 62.04 (95% CI: 59.94, 64.14) and 42.57 (95% CI: 40.36, 44.78), respectively.

**Conclusion:** Although a relatively acceptable knowledge was detected in this high-risk population, practices regarding TB and HIV showed some weaknesses. Developing special programs to improve the healthy behavior of this population is highly recommended.

## Introduction

The acquired immunodeficiency syndrome (AIDS) has become a pandemic threat to the world population.^1^ After the introduction of the HIV in 1983,^2^ the disease was first reported in Iran (a boy with hemophilia) in 1986. Based on national reports, a total of 34 846 HIV-infected people have been identified in Iran until December 2016. However, according to the estimates, there are over 76 000 people living with HIV in Iran. The most predominant method of HIV transmission in Iran is injection drug use, with sexual contact has been recently acknowledged as an important mode of transmission. Educational programs have been introduced as an important tool to HIV prevention.^3^

HIV is the most significant known risk factor for activation of latent tuberculosis infection (LTBI). The risk of active tuberculosis (TB) for people with both LTBI and HIV reaches 8% to 10% per year, whereas this same risk for people without HIV infection is 5% to 10% in a lifetime.^4,5^ It has to be noted that TB may be the first indicator of HIV infection.^6,7^

Factors such as low income, living and working in substandard conditions, limited access to nutrition and proper healthcare services predispose people to diseases like TB. Occupations such as miners, construction workers, and health care workers, and prisoners and people who live and work in improper conditions have a higher risk of TB.^8^

Homeless people are individuals who either have no place to sleep or sleep in public or private shelters. An estimated 100 million people are homeless worldwide. The number of homeless is increasing in Tehran and other big cities of Iran, as 4811 homeless were registered in Eslamshahr center in 2008-2009.^9^ The number of registered homeless females rose during 2006-2011.^10^ The fatality rate among this group of people is almost 4 times higher than that of the general population.^11^

Homelessness is associated with social, behavioral, and environmental hazards that make homeless people vulnerable to communicable diseases and make a public health concern. Due to their specific lifestyle, high-risk behaviors such as drug use, needle sharing, and selling sex, HIV risk is higher in homeless people.^3,12-14^ It seems that homelessness can potentially rise due to increasing impulsive behaviors.^15^

Also, these groups are increasingly at risk for exposure to *Mycobacterium tuberculosis* and consequently develop active TB with a co-infection of TB and HIV.^16,17^ According to an unofficial report, more than 18 000 homeless are living in Tehran. One useful way to decrease undesirable consequences induced by infectious diseases is to improve the health interventions.

Understanding the baseline status of knowledge, attitude, and practice (KAP) in the target population is necessary. Although there are some studies conducted in Iran concerning the KAP of various high-risk groups,^18-20^ homeless people have not been targeted yet. The present study was conducted to define the KAP of this population in Tehran regarding TB and HIV.

## Methods

### Study Population

We conducted a cross-sectional study on the homeless people in five centers sponsored by the municipality of Tehran from June to August 2012. These centers are the whereabouts of homeless people in Tehran that collect this group from the corners of streets, in parks, or in public places and keep them temporarily.

In this study, a homeless person is defined as someone who had no home or shelter to reside in and instead resides on the corners of streets, in parks, or in public places, if he/she is not provided with a residence provided by the governmental or non-governmental organizations.^11,12^ Inclusion criteria of this study are the willingness to participate in the study, having an age of 18 to 60 years, and being homeless for at least 10 days during the month prior to the study period either continuously or intermittently. Exclusion criteria, on the other hand, are a reluctance to participate in the study and participation in another similar study.

### Data Collection

In this study, a bio-behavioral survey among homeless people was employed. It is worth mentioning that the results of the survey regarding the prevalence of communicable disease have been published elsewhere.^21^

Before approve approved, several meetings were held by the municipality, state welfare organization of Tehran, and Social Security police. Through these meetings, the researchers introduced the main service provider centers for homeless in Tehran. The Municipality of Tehran provided shelters, a bed, two meals a day, health services, and counseling for homeless people.^21^ They could enter and exit freely only in two centers (Bahman and Khavaran shelters) while in the other centers homeless are brought by the police force and had prohibition of exit until spending courses and specific terms. Sampling was done in five centers including Khavaran, Bahman, Lavizan, and EslamShahr shelters, and Shafagh camp.

Since there was no specific questionnaire designed for homeless people, a questionnaire in Persian was developed to achieve the required information. For this purpose, some previous body of evidence in Iran^20,22^ and related indicators in international reports were reviewed to address more important issues in high-risk populations.

The questionnaire consists of six sections as follows: (*a*) demographic characteristics; (*b*) six questions related to knowledge of HIV (one point for each question) with a total score between zero and six; (*c*) three five-choice questions related to attitude toward HIV (strongly disagree = 5, disagree = 4, no idea = 3, agree = 2, and strongly agree = 1) with a total score between zero and 15; (*d*) two questions related to practice (one point for each question) regarding HIV with a total score between zero and two; (*e*) five questions related to knowledge of TB (one point for each question) with a total score between zero and five; and (*f*) two questions about practice regarding TB (one point for each question) with a total score between zero and two. Each HIV and TB KAPs score was divided by the maximum score and multiplied by 100 to obtain percentage scores.

A panel of an expert in the field of TB and HIV epidemiology evaluated the validity of the questionnaire. The reliability of the questionnaire (ie, 0.69) was also investigated by the test and retest technique with an interval of one week. Interviews and questioning were conducted by trained interviewers. All participants were screened if they had heard anything about TB or HIV. Next, KAP questions were filled by people who had heard anything about TB or HIV.

### Statistical Analysis

An analysis of variance (ANOVA) and a student’s *t* test were used to compare the mean score of KAP across subgroups. A Tukey test was used for post hoc analysis and two-by-two comparisons. All analyses were performed at a 95% confidence level using SPSS 16.

## Results

In this study, a total of 593 participants were interviewed; the mean ± standard error of mean (SEM) age of the participants was 41.74 ± 10.77 years, males were significantly older (42.74 ± 10.56) than females (35.46 ± 9.96) (*P* = .001). The mean duration of homelessness was 50.71 ± 78.17 months. Study participants were predominantly males (86.51%) who had a more homelessness duration (50.71 ± 78.18 months) compared to females’ homeless duration (12.68 ± 25.87 month) (*P* = .001). Basic characteristics of the recruited homeless subjects are shown in Table 1.

**Table 1:** Basic Demographic Characteristics of Homeless People of Tehran, 2012

**Variable**	**No. (n = 593)**	**%**
Gender		
Female	80	13.49
Male	513	86.51
Age group		
18-29	85	14.62
30-39	175	30.12
40-49	138	23.75
≥50	183	31.50
Marriage		
Single	117	24.48
Married	71	14.85
Divorced/widowed	290	60.67
Education		
Illiterate	68	11.83
Can read and write/primary school	145	29.74
Secondary school	155	26.96
High school/diploma	155	26.96
Post high school	52	9.04
Homelessness duration		
10 days-5 months	146	25.70
5-24 months	171	30.11
24-60 months	118	20.77
≤60 months	133	23.42
History of incarceration		
Never	298	51.20
More than 10 years ago	69	11.86
Within the last 10 years	215	36.94
Duration of incarceration (in the last 10 years)		
More than 11 months	107	50.23
Less than 11 months	106	49.77
Using condom in last sexual contact	154	33.19
History of sharing needles	64	52.46
History of having sex with males among males	36	7.02
History of selling sex among women	14	17.50
Smoking cigarettes regularly^a^	507	85.49
Smoking hookah	108	18.21

^a^ Currently using cigarettes and smoking every day.

### Knowledge, Attitude, and Practice About HIV

Only 395 participants (70.30% of males and 50% of females) had heard something about HIV while 28.42% of them considered themselves at risk for HIV (Table 2). In this regard, there was no significant difference between males and females (*P* = .43).

**Table 2:** Descriptive Analysis Regarding General HIV and TB Knowledge and Attitude of Homeless People in Tehran, 2012

**Question**	**No. (%) of Positive Answers**
HIV	
Have you ever heard anything about HIV/AIDS?	395 (66.61)
Knowledge about HIV	
Can using condoms reduce the risk of HIV transmission?	323 (82.39)
Can a healthy looking person have HIV?	296 (75.70)
Can a person get HIV from mosquito bites?	197 (49.87)
Can a person get HIV by sharing a meal with someone who is infected?	229 (57.97)
Can HIV be transmitted via sharing syringes or needles with others?	369 (93.89)
Can HIV be transmitted via sex between two men?	367 (93.15)
Attitude toward HIV	
Do you consider yourself at risk of HIV?	110 (28.42)
HIV positive people should be isolated from the general population.	206 (52.41)
The presence of an HIV positive person makes a family embarrassed.	157 (40.67)
Would you share a meal with a person who is positive for HIV/AIDS?	163 (42.12)
TB	
Have you ever heard anything about TB?	343 (58.73)
Knowledge about TB	
Does TB spread by respiratory route?	296 (86.30)
Can a person get TB by sharing a meal with someone who is infected?	122 (35.67)
Can TB be transmitted via sharing syringes or needles with others?	122 (35.88)
Does living with a TB infected person raise the risk of getting a TB infection?	279 (83.80)
Is TB a treatable disease?	245 (72.70)
Do you consider yourself at risk of TB?	126 (36.95)
Practice regarding TB	
Smoking cigarettes regularly	507 (85.49)
Smoking hookah	108 (18.21)

Abbreviation: TB, tuberculosis.

The total mean score of knowledge toward HIV was 79.24 (95% CI: 77.36, 81.12). The mean score of knowledge among males (80.42) was more than that of female (68.75) significantly (*P* = .001). Compared the illiterate people, a significantly higher knowledge of HIV was detected in people with the academic education (*P* = .02). The mean score of attitude in educational groups was significantly different (*P* < .001) and people with an elementary level of education had the lowest score of attitude. The mean score of attitude toward HIV was different in terms of marital status (*P* = .03) as it was higher in single people compared to the married ones. The total mean score of practice toward prevention of HIV was 21.14 (95% CI: 18.35, 23.93). However, practice score among males was significantly less than females (*P* = .001). In addition, in post hoc analysis, the score of practice among age groups was different (*P* = .045) and people aged 41-51 years had a higher score compared to those over 51 years (Table 3).

**Table 3:** Distribution of KAP Mean Score Percentages for Participants Who Heard About HIV/AIDS, Tehran, 2012^a^

**Variable**	**N**	**Knowledge**	***P *** ** value**	**Attitude**	***P *** ** Value**	**Practice**	***P *** **Value**
		**Mean (95% CI)**		**Mean (95% CI)**		**Mean (95% CI)**	
Gender							
Female	40	68.75 (63.37, 74.13)	.001	54.67 (48.61, 60.73)	.42	35.00 (27.00, 43.00)	.001
Male	355	80.42 (78.45, 82.39)		57.41 (55.28, 59.54)		19.58 (16.65, 22.51)	
Age group							
18-29	50	74.67 (69.58-79.75)	.06	59.60 (53.70-65.50)	.48	24.00 (15.76-32.24)	.004
30-39	129	79.06 (75.66-82.48)		56.33 (52.94-59.72)		21.32 (16.48-26.16)	
40-49	86	83.53 (79.95-87.10)		59.46 (54.4-64.27)		29.07 (22.00-36.14)	
≥50	126	78.57 (74.99-82.15)		55.87 (52.37-59.38)		15.08 (10.86-19.29)	
Marital status							
Single	82	80. 08 (10.51, 19.89)	.31	61.54 (57.14, 65.94)	.03	28.05 (21.23, 34.87)	.30
Married	46	77.90 (73.59, 82.21)		50.58 (44.44, 56.72)		26.09 (18.18, 34.00)	
Divorced	184	78.71 (75.94, 81.43)		58.33 (55.49, 61.17)		21.47(17.34, 25.60)	
Widowed	23	86.23 (79.84, 92.98)		60.57 (52.07, 60.38)		19.57 (6.96, 32.17)	
Education							
Illiterate	29	74.71 (67.71, 81.71)	.001	51.26 (44.97, 57.55)	.001	17.24 (7.18, 27.30)	0.32
Primary	77	67.75 (63.46, 72.04)		48.14 (43.95, 52.33)		16.23 (10.66, 21.80)	
Secondary school	105	81.59 (78.23, 84.95)		58.92 (55.27, 62.57)		20.48 (14.90, 26.06)	
High school/diploma	129	82.04 (78.83, 85.25)		59.69 (56.02, 63.36)		23.26 (18.31, 28.21)	
Post high school	47	87.59 (83.58, 91.60)		62.55 (56.81, 68.29)		25.53 (16.65, 34.41)	
Homeless duration (months)								
5-10 days	85	79.80 (76.01, 83.59)	.50	56.16 (51.79, 60.53)	.52	22.94 (16.47, 29.41)	0.75
5-24	124	81.18 (77.79, 84.57)		56.13 (52.56, 59.70)		20.97 (16.33, 25.61)	
24-60	82	77.44 (73.66, 81.22)		58.46 (54.22, 62.70)		22.56 (16.16, 28.96)	
≤60	96	78.30 (74.31, 82.29)		58.96 (54.70, 63.22)		18.75 (13.08, 24.42)	
History of incarceration							
Never	173	76.69 (73.64, 79.74)	.07	57.96 (54.98, 60.94)	.07	19.65 (15.74, 23.56)	0.64
More than 10 years ago	52	81.42 (77.06, 85.78)		50.90 (45.61, 56.19)		22.11 (13.85, 30.37)	
Within the last 10 years	167	81.14 (78.36, 83.92)		58.00 (54.83, 61.17)		22.46 (17.93, 26.99)	
Duration of Incarceration (in the last 10 years)							
More than 9 months	85	80.39 (76.60, 84.18)	.60	57.41 (53.00, 61.82)	.72	21.18 (15.18, 27.18)	0.63
Less than 9 months	81	81.89 (77.74, 86.04)		58.60 (53.95, 63.25)		23.46 (16.55, 30.37)	
Total	395	79.24 (77.36, 81.12)	-	57.13 (55.12, 59.14)	-	21.14 (18.35, 23.93)	-

Abbreviation: KAP, knowledge, attitude, and practice. ^a^ Participants’ scores of KAP are calculated out of 100.

In this study, 82.39% of the participants knew condoms as a preventive tool for protection against HIV and 35.63% of them had used this tool in their last sexual intercourse. Males had more knowledge about the preventive role of condoms in HIV transmission. On the other hand, women reported significantly more condom use in their last sexual intercourse (69.44% vs. 31.10%, *P* < .001).

Over 90% of participants knew needle sharing is one method of HIV transmission and 13.62% of them had a history of needle sharing.

Totally, 93% of participants knew the possibility of HIV transmission through unprotected sex between men. Of 367 males who answered correctly to this question, 29 (7.90%) had experienced sex with other men. Generally, 14 women (17.50%) reported prostitution in their lifetime, and 11 (78.57%) of them had heard about HIV.

### Knowledge, Attitude, and Practice About Tuberculosis

In this study, 343 (57.84%) participants (62.18% of males and 36.70% of females, *P* < .001) had heard about TB and 36.95% of them considered themselves at risk of TB. In this regard, there was no significant difference between males and females (*P* = .22) (Table 2).

The total mean score of knowledge about TB was 62.04 (95% CI: 59.94, 64.14).

Post hoc analysis showed that people with a high education had more knowledge of TB in comparison to illiterate people (*P* = .002), but no significant difference was detected with other education levels. The overall mean score of practice toward TB was 42.57 (95% CI: 40.36, 44.78). The mean score of practice among males was more than that of females (*P* = .001) (Table 4).

**Table 4:** Distribution of percent KAP mean scores for participants who heard about TB, Tehran 2012

**Variable**	**N**	**Knowledge**	***P*** ** Value**	**Practice**	***P*** ** Value**
		**Mean (95% CI)**		**Mean (95% CI)**	
Gender					
Female	29	61.37 (52.26, 70.48)	.85	27.58 (18.37, 36.79)	.002
Male	314	62.10 (59.96, 64.24)		43.94 (41.74, 46.14)	
Age group					
18-29	39	64.62 (57.11-72.12)	.64	37.18 (28.29-47.07)	.11
30-39	91	63.52 (59.10-67.63)		40.11 (35.66-44.56)	
40-49	81	61.23 (57.14-65.33)		45.68 (41.69-49.66)	
≥50	129	60.93 (59.99-64.24)		43.41 (40.07-44.56)	
Marital status					
Single	70	64.00 (59.09, 68.91)	.84	36.43 (30.04, 42,82)	.09
Married	47	61.70 (55.60, 67.80)		42.55 (37.27, 47.83)	
Divorced	159	62.14 (59.05, 65.22)		44.03 (40.93, 47.13)	
Widowed	19	65.26 (56.26, 74.26)		44.74 (37.14, 52.33)	
Education					
Illiterate	30	56.00 (49.09, 62.91)	.001	41.67 (34.59, 48.74)	.12
Primary	70	56.57 (51.98, 61.16)		43.57 (38.23, 48.91)	
Secondary school	82	60.73 (56.39, 65.07)		38.41 (33.75, 43.08)	
High school/diploma	109	63.85 (60.07, 67.39)		42.67 (38.61, 46.71)	
Post high school	45	70.67 (65.29, 76.04)		48.89 (43.83, 53.94)	
Homeless duration (months)					
5-10 days	70	63.71 (58.19, 62.24)	.71	40.00 (34.79, 45.21)	.38
5-24	101	60.59 (56.83, 64.36)		40.59 (36.47, 44.71)	
24-60	79	62.78 (57.87, 66.70)		43.67 (39.51, 47.93)	
≤60	86	60.93 (56.78, 65.08)		44.77 (40.11, 49,43)	
History of incarceration					
Never	157	60.38 (57.08, 63.68)	.33	41.40 (37.92, 44.88)	.66
More than 10 years ago	42	63.81 (57.93, 69.69)		44.05 (37.89, 50.21)	
Within the last 10 years	141	63.54 (60.38, 66.71)		43.26 (39.93, 45.60)	
Duration of Incarceration (in the last 10 years)					
More than 9 month	73	61.92 (57.52, 66.32)	.33	41.78 (37.09, 46.47)	.38
Less than 9 month	67	65.07 (60.55, 69.59)		44.78 (40.06, 49.50)	
Total	343	62.04 (59.94, 64.14)		42.57 (40.36, 44.78)	

Abbreviations: KAP, knowledge, attitude, and practice; TB, tuberculosis. ^a^ Participants’ scores of knowledge and practice are calculated out of 100.

Only 301 participants (50.76%) had heard about both HIV and TB, of whom 14 (4.65%) subjects had comprehensive knowledge about both HIV and TB.

## Discussion

In this study, 66.61% and 58.73% of the participants declared that they had heard about HIV and TB, respectively. This percentage was lower for other groups considered at risk for HIV (female sex workers, prisoners, and injection drug users [IDU]) in Tehran.^20^

In this study, only 66.61% of homeless participants have heard about HIV/AIDS that is much lower than the results of the surveys on female sex workers (FSWs) (92.70%), IDUs (96.93%), and prisoners (93.19%).^20^ This result shows that although education programs about the communicable disease for high-risk people such as FSWs, IDUs, and prisoners have been recently improved in Iran, it still covers a low population of homeless.

These results indicate a gap between the scores of knowledge and practice among participants regarding HIV and TB. The results of our study indicate a higher mean score of knowledge about HIV (79.24) than the results of a meta-analysis (67.51) conducted in Iran.^23^ In a cross-sectional study in 13 provinces of Iran on young people, less than 40% of participants had a high knowledge score about HIV.^22^ Participants in our study may have received further information about HIV through educational programs or counseling in jails, shelters, addiction treatment centers, etc. A reason for a gap between scores of knowledge and practice regarding HIV may be due to the lack of discretion in the use of a condom, especially among female homeless people. On the other hand, some factors such as lack of social and emotional support, and economic poverty among homeless people could affect the behaviors of these people. Another reason for this difference may be the lower number of knowledge questions in our study.

In this study, the negative attitude of the participants about HIV was consistent with another study.^24^ Over 90% of participants in our study were aware of the risk of HIV transmission via contaminated syringes. The awareness of this transmission method is common in Iran because of the design and implementation of educational programs for different groups in recent years. Similar to our study, most studies have results over 80% of participants knowing the transmission methods of HIV.^18,20,25,26^

In our study, about 93% of participants knew homosexual intercourse as a risk factor for transmission of HIV. Although 36 (7.02%) of all male participants reported a history of homosexual contact, considering the stigmatized nature of this behavior in our public opinion that may lead to refuse disclosure, the real prevalence of this behavior might be higher among this high-risk population.

Contrary to the high mean score of knowledge about HIV among men (*P* = .001), the mean score of practice was significantly lower for males (*P* = .001). A reason for this difference may be due to significantly more condom use in females compared to the males in their last intercourse. These results may indicate that, despite the lower knowledge of women, they are more cautious about their health than men. It can also be explained by the fact that women may use a condom for prevention of unwanted pregnancy without knowing that using condoms prevents HIV transmission.

In line with some similar studies,^19^ the knowledge level of men about the preventive role of condoms in the prevention of HIV was higher than that of women. Condom use was also more reported among homeless African-American female teenagers than men^27^ while another study on Mexican adolescents indicated the opposite of this finding.^28^

The mean score of practice among men, however, was lower than that of women. An explanation for this result may be the higher mean age of men than women, as condom use often decreases with increasing age.^11^ Inconsistent with this finding, in a qualitative study on socially damaged women, it was found that the main reason for having unprotected sex by 88% of the participants was lack of authority to make decisions on the sexual contacts and use of condoms.^29^ Generally, in our study, the frequency of condom use among homeless people was low, as indicated in other high-risk groups.^20^ The main reasons for this practice, especially in Iran, maybe unavailability and cost issues.^30^ Most people who sell sex (either male or female) to obtain goods or drugs or women who are forced into the sex trade by their addicted relatives, do not have a choice in using condoms, as this decision usually remains by their sexual partners.^29,31,32^

The mean score of TB knowledge in this study (62.04) was higher than that of the Los Angeles homeless (57.31).^33^ Although there was no significant difference between knowledge of men and women regarding TB, the mean score of practice about TB among men was significantly higher than that of women. The significant impact of knowledge and practice regarding TB on effective control of this infection among homeless is clear.^34^ Poor knowledge about TB may foster treatment delays.^35^

In this study, knowledge of educated people was high toward HIV and TB. There was, though, no significant difference in practice toward HIV and TB among people with different levels of education.

Therefore, it is implied that having the knowledge and even changing attitude does not necessarily lead to change in practice. For effective impact on all three fields, we need more comprehensive educational programs. The risk of HIV should be tangible for homeless people with high-risk behaviors. In the educational programs, the volunteer people living with HIV and AIDS can have an effective role with their peer support.^36^

This study had limitations for the number of required questions to complete evaluation of HIV and TB KAP. Such limitations are attributed to fact that the original study was so wide and its primary aim was to assess the prevalence of HIV, viral hepatitis, and TB among homeless people. Another limitation of this study is that there was only one question to assess the attitude of the participants about TB in the original questionnaires.

Moreover, this study was a facility-based survey and it was not possible to include the homeless people who had not been engaged in shelters. Thus, we should be cautious to generalize the results to all homeless population.

## Conclusion

The results of our study showed a major gap among KAP regarding HIV and also between knowledge and practice toward TB. Rescuing street children, improving their living conditions, and providing educational programs can prevent them from committing high-risk behaviors in the future. Also, educational and counseling programs in prisons, harm reduction centers, and shelters can be useful to prevent from high-risk behaviors in these groups.

## Acknowledgments

Our special thanks go to the research committee and the ethical committee of the Pasteur Institute of Iran, Tehran, Iran which approved this project (No. 1716) and the Center for Communicable Disease Control in the Ministry of Health and Medical Education (MoHME), Tehran, Iran which financially supported this project. The research team is grateful for the technical support of Dr. Ali Akbar Haghdoost (WHO Collaboration Center, Regional Knowledge Hub for HIV/AIDS Surveillance, Kerman, Iran), for the logistical support of Dr. Saber Esmaeili (Pasteur Institute of Iran, Tehran, Iran), Dr. Zahra Bolouki (Tehran University of Medical Sciences, Tehran, Iran) and Dr. Fereshteh Ansari (University of Tehran, Tehran, Iran) and all the Tehran municipality centers which helped us with the sampling process. This project was supported by Centre for Communicable Disease Control in the MoHME, Tehran, Iran (Grant No. 1716).

## Ethical issues

This study was approved by ethical committee of Pasture Institute of Iran, Tehran, Iran (No. 1716).

## Competing interests

Authors declare that they have no competing interests.

## Authors’ contributions

FBA, AD, and EM obtained the data, performed analyses, and wrote results. EM, FBA, and AS took a lead in review of literature. All five authors contributed to discussion and conclusions.

## Authors’ affiliations

^1^Urology and Nephrology Research Center, Shahid Beheshti University of Medical Sciences, Tehran, Iran. ^2^Department of Epidemiology, School of Public Health, Hamadan University of Medical Sciences, Hamadan, Iran. ^3^Department of Epidemiology and Biostatistics, Research Centre for Emerging and Reemerging Infectious Diseases, Pasteur Institute of Iran, Tehran, Iran. ^4^Blood Transfusion Research Center, High Institute for Research and Education in Transfusion Medicine, Tehran, Iran. ^5^Center for Communicable Disease Control, Ministry of Health and Medical Education, Tehran, Iran. ^6^Department of Epidemiology and Biostatistics, School of Public Health, Tehran University of Medical Sciences, Tehran, Iran. ^7^HIV/STI Surveillance Research Center, and WHO Collaborating Center for HIV Surveillance, Institute for Futures Studies in Health, Kerman University of Medical Sciences, Kerman, Iran.

## Key messages

**Implications for policy makers**

- Educational and counseling programs in prisons, harm reduction centers, and shelters can help governors prevent high-risk behaviors in homeless people.
- A concerted national strategy is needed to decrease the size of the homeless population.

**Implications for the public**

Homeless people are more vulnerable to infectious diseases due to their poor nutrition, high-risk behavior, and low knowledge. The relation between homeless people and the general population is documented, especially in the area of HIV. These connections may result in transmission of diseases such as HIV to low-risk populations. Understanding the baseline status of knowledge, attitude, and practice (KAP) in homeless people can help the policymakers to find the gaps and develop specific programs accordingly.
